# Automated Detection of Acute Lymphoblastic Leukemia From Microscopic Images Based on Human Visual Perception

**DOI:** 10.3389/fbioe.2020.01005

**Published:** 2020-08-28

**Authors:** Alexandra Bodzas, Pavel Kodytek, Jan Zidek

**Affiliations:** Department of Cybernetics and Biomedical Engineering, Faculty of Electrical Engineering and Computer Science, VSB-Technical University of Ostrava, Ostrava, Czechia

**Keywords:** automated leukemia detection, blood smear image analysis, cell segmentation, leukemic cell identification, acute leukemia, image processing, machine learning

## Abstract

Microscopic image analysis plays a significant role in initial leukemia screening and its efficient diagnostics. Since the present conventional methodologies partly rely on manual examination, which is time consuming and depends greatly on the experience of domain experts, automated leukemia detection opens up new possibilities to minimize human intervention and provide more accurate clinical information. This paper proposes a novel approach based on conventional digital image processing techniques and machine learning algorithms to automatically identify acute lymphoblastic leukemia from peripheral blood smear images. To overcome the greatest challenges in the segmentation phase, we implemented extensive pre-processing and introduced a three-phase filtration algorithm to achieve the best segmentation results. Moreover, sixteen robust features were extracted from the images in the way that hematological experts do, which significantly increased the capability of the classifiers to recognize leukemic cells in microscopic images. To perform the classification, we applied two traditional machine learning classifiers, the artificial neural network and the support vector machine. Both methods reached a specificity of 95.31%, and the sensitivity of the support vector machine and artificial neural network reached 98.25 and 100%, respectively.

## Introduction

Leukemia is a term describing a group of hematological malignancies that are manifested by the tumourous proliferation or increased life span of immature white blood cells (WBCs) in the bone marrow (American Dental Association [ADA], 2012). Leukocytes are highly differentiated for their specialized functions, and they play an essential role in the immune system (Rogers, 2011). The malignancy of this disease varies from non-malignant to highly aggressive forms, and the immature cells are not able to fulfill their normal function (Serfontein, 2011). The excessive production of these type of cells, denoted as blasts or leukemic cells crowds out healthy leukocytes in the bone marrow and suppresses normal hematopoiesis, causing difficulties in fighting infections, transporting oxygen and controlling bleeding (Daniels and Nicoll, 2012). Clinically, leukemia is categorized on the basis of the rapidity of the disease progression to acute and chronic forms. Whereas the acute form of leukemia develops quickly and the number of leukemic cells increases rapidly, chronic leukemia progresses slowly over time, and the more mature leukocytes can carry out some of their normal functions (Serfontein, 2011). According to the type of affected cell from which the malignancy develops, leukemia is further divided into myelogenous and lymphoid forms (Manisha, 2012). Acute lymphoblastic leukemia (ALL), which is the only form we consider in this paper, is the second most common type of leukemia in adults and the most common type of childhood malignancy, accounting for approximately one-third of all pediatric cancers (Rose, 2013). Heterogeneous malignancy is caused by genetic alterations and chromosomal mutations of lymphocyte progenitor cells at an early phase of cell differentiation (Rose, 2013). The excessive production of these cells, called lymphoblasts, which do not develop into mature B and T lymphocytes, gradually displaces normal cells in the bone marrow and may spread to essential organs such as the liver, lymph nodes, spleen, and central nervous system (Katz et al., 2015).

The diagnosis of ALL requires a broad spectrum of information derived from several modalities, including morphology, cell phenotyping, cytochemistry, cytogenetics, and molecular genetics (Inaba et al., 2013). Despite technological advances in medicine, morphology remains the frontline hematological diagnostic technique. The observation of excessive leukemic cell buildup and morphological anomalies in cellular structures during the visual examination of peripheral blood smears arouses the first suspicion of leukemia. Because manual microscopic examination is a time-consuming process that requires a considerable amount of experience and is prone to humane error (Inaba et al., 2013), such an automated inspection is needed, which would standardize the examination process and circumvent the drawbacks of this diagnostic technique.

To minimize human intervention and overcome the abovementioned limitations, several computerized methods have been explored. Most of these methods utilize conventional image processing and machine learning techniques, which involve mainly segmentation, feature extraction, and classification methods. Especially the segmentation and feature extraction phases are considered the most significant and challenging tasks (Neoh et al., 2015). The main reason lies in the large variety of blood smear images, taken under different conditions, and the potential morphological differences between blast cells. Although some of these proposed methods were found to be faster and more cost effective than manual examination, their impact and accuracy remain insufficient (Shafique and Thesin, 2018). Whereas, Wang et al. (2019) achieved a detection speed of 14 to 100 milliseconds by utilizing convolution neural networks and GPU, most proposed methods produce false-negative errors and achieve overall accuracy in the range of 93–98% (Bagasjvara et al., 2016).

In this study, we propose a novel combination of techniques to overcome the most challenging parts of the detection process and present detailed insights into the greatest shortcomings of the existing classification methodologies, such as the overfitting and the reliability of particular classifications. To improve our segmentation phase, we introduce extensive pre-processing based on the proposed color transformation and design a three-phase filtration that ensures the elimination of surrounding blood components and artifacts without disrupting particular regions of leukocytes. After the whole segmentation process, involving seven stages, a robust set of features is extracted from all segmented regions. Extracting morphological and texture features from specific cell regions in a similar way to the visual interpretation of a domain expert heightens the performance of the selected classifiers. The final recognition of ALL from peripheral blood smear images is accomplished by an artificial neural network (ANN) and optimized support vector machine (SVM).

## Literature Review of the Previously Proposed Methodologies

Extensive research has recently been conducted to explore the possibilities for the automated detection of leukemia from microscopic blood smear images (Alsalem et al., 2018). Most previously proposed methods employ sequential image pre-processing, cell segmentation, feature extraction, and cell classification (Bodzas, 2019). The main aim of the pre-processing phase is to enhance the image quality for subsequent processing. Many authors have enhanced blood smear images by converting them to another color domain, which highlights the particular features of the objects and therefore increases the efficiency of region detection (Aljaboriy et al., 2019). For example, Putzu et al. (2014) and Hariprasath et al. (2019) stated that the identification of WBCs is possible with conversion to the CMYK color model. The reason is that leukocytes have a higher contrast in the Y component since the yellow color is present in all elements except WBCs.

On the other hand, Moradiamin et al. (2015) converted images from the RGB color space to HSV, which reduced the correlation between the color channels in comparison to RGB and enabled the three H, S, and V channels to be dealt with separately. They additionally complemented this with a pre-processing phase with histogram equalization, which reduced the effect of different lightening conditions. After nucleus segmentation by the fuzzy C-means clustering algorithm, the authors extracted five geometrical and 72 statistical features. The dimensionality of the feature set was reduced by principal component analysis to eight features, which were subsequently applied to the SVM classifier.

A different approach was introduced by Kazemi et al. (2016) by implementing selective median filtering in combination with conversion to the CIEL^∗^a^∗^b model, in which the perceptual difference between colors is proportional to the Cartesian distance. In simple terms, the formula CIEL^∗^a^∗^b takes the XYZ tristimulus values and the white reference to produce correlates to the luminence, chroma, and hue elements (Fairchild, 2005). To extract the nucleus of WBCs, color-based clustering segmentation with additional morphological filtering was implemented. The set of features, including irregularity, the Hausdorff dimension, shape, color, and texture, was extracted from a whole image containing multiple nuclei. By applying a two-class SVM, they were able to achieve an overall accuracy of 96%.

In addition to the clustering segmentation method, many authors have used thresholding-based techniques to segment WBCs. In particular, Joshi et al. (2013) reported the usage of Otsu’s global thresholding on an enhanced greyscale image. To differentiate blasts in a microscopic blood smear image, they extracted the area, perimeter, and circularity from the equivalent binary image and employed the K-nearest neighbor decision algorithm for classification.

Due to the absence of spatial information, threshold techniques cannot always produce relevant and precise results. Hence, they are often combined with mathematical morphology or other image processing techniques. For instance, Wang et al. (2008) proposed a segmentation algorithm that combined adaptive thresholding with an edge-based technique and seeded watershed to recognize cell nuclei in different cycle phases. Moreover, unlike other studies using off-line learning algorithms, the authors in this study deployed an online SVM classifier, which removed the support vectors from the older model and assigned weights to the new samples according to their importance to accommodate changing conditions.

Concerning feature extraction and classification, recent research has shown that the most preferred methodologies use a combination of morphological and texture features with supervised learning algorithms. In particular, SVM and multilayer perceptron have provided higher accuracy than methods using other classifiers (Aljaboriy et al., 2019). For instance, research by Neoh et al. (2015) extracted a total of 80 feature descriptors containing color, shape, and texture information to compare the classification performance of the SVM and multilayer perceptron. Both classifier results reached a similar accuracy, over 95%, with slightly higher accuracy for the multilayer perceptron classifier.

## Materials and Methods

The main goal of this work is to develop a fully automated system for ALL detection that can be applied to complete blood smear images containing multiple WBCs. The solution presented in this paper is based on conventional image processing techniques and comprises four main stages, which are described in the following subchapters.

### Blood Smear Image Dataset

The proposed system was trained as well as tested on a local dataset, which was provided by the Department of Haemato-oncology at the University Hospital Ostrava. The anonymized dataset consists of 18 microscopic blood smear images obtained from patients without pathological findings and 13 blood smear images from patients with diagnosed ALL. On average, six blood smear images with a resolution of 4,080 × 3,072 were captured per patient. Since WBCs are distributed unevenly, with a predominance of large cells on the border and smaller cells in the center of the blood smear, systematic data acquisition was required (Bodzas, 2019, p. 45). This was carried out by the meander inspection pattern, which allowed microscopic images to be captured from different consecutive locations, particularly from both edges and the center of the blood smear. All slides in the dataset were stained with Giemsa stain and were captured under the same lighting conditions by an Olympus CX43 microscope under a magnification of 50 times with an oil immersion objective lens and an effective magnification of 500 (Bodzas, 2019, p. 45).

The manual examination of blood smear images was conducted by local domain experts. During this visual examination, the hematology specialists used several morphological criteria to distinguish between lymphoblasts and normal cells. The most significant criteria included the nucleus position and shape, chromatin structure, presence of nucleoli, nucleocytoplasmic ratio, size of the cell, and color or structure of the cytoplasm. Following the WHO classification system, ALL is divided into B-lymphoblastic leukemia/lymphoma, T-lymphoblastic leukemia/lymphoma, and acute leukemias of ambiguous lineage. Because, from a morphological point of view, there are no reproducible criteria to distinguish between B and T lineage lymphoblastic leukemia, ALL subtype classification is not considered in this study (Chiaretti et al., 2014).

### Pre-processing

During the acquisition process, numerous variable factors, such as different illumination conditions, staining time, blood film thickness and film defects, may introduce undesirable visual artifacts or cause different color distributions among the images (Díaz and Manzanera, 2009). To deal with potential microscopic image artifacts and enhance the contrast of the individual blood elements, we introduced a pre-processing method based on the standard arithmetic operations followed by gamma correction and contrast enhancement algorithms. The proposed color transformation is described by the following formula

(1) g⁢(x,y)=[(L-1)-B]-{[(L-1)-G]⁢0.5}

where *g*(*x*, *y*) is the transformed image, *L* is the number of distinct gray levels in the image and *B* and *G* are the blue and green color spaces. Using arithmetic operations on the individual color spaces enhanced the blood smear images and allowed finer differentiation of the leukocytes, even for cells with scanty cytoplasm (Bodzas, 2019, p. 46).

### Leukocyte Segmentation

**FIGURE 1:**
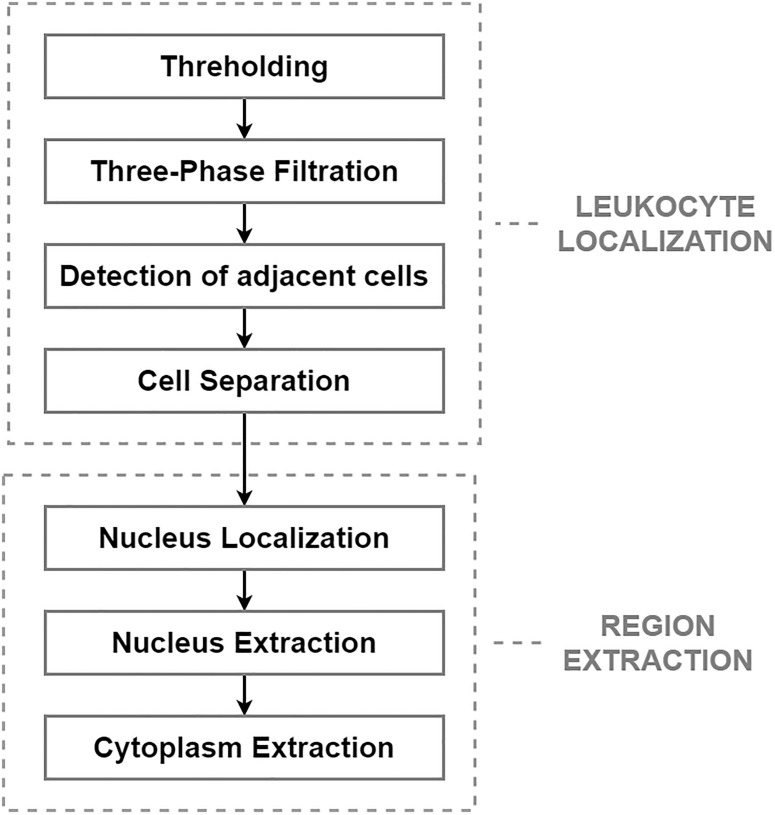
The proposed segmentation algorithm.

After applying the pre-processing step, the segmentation phase was performed. The segmentation phase, which is concerned with extracting individual object components carrying pivotal information, is considered the most essential and challenging task. The aim of this task is to reduce the computational complexity of the subsequent steps, and to reduce the size of the high-resolution images, which heavily burden the storage capacity of the hospital’s server (Chen et al., 2020). From a morphological point of view, leukemic cells can be distinguished from mature leukocytes by having a large nucleus with finely dispersed chromatin, moderate and non-granular cytoplasm, and one or more prominent nucleoli (Wiernik, 2001). The challenging process in this work comprises two main steps: leukocyte localization and region extraction, which separates the specific cell components (nucleus and cytoplasm). The entire segmentation process, divided into these two main parts, is shown in Figure 1.

The most precise segmentation results of the leukocyte localization phase were achieved by an algorithm involving four fundamental stages, which can be seen in the diagram above. The main aim of this phase was to remove the background and the surrounding blood components and to separate any touching cells. The first step of this challenge is the conversion of the image into a binary format, which was performed by the histogram-based thresholding segmentation method. Due to the sensitive pre-processing phase, thresholding reduced the background and part of the erythrocytes, while the full size of the WBCs was retained. Considering that erythrocytes usually have the shape of a biconcave disk with an inclination to overlap each other and that platelets lie in a different color spectrum, the process of thresholding often results in an image with additional noise. To eliminate the residual parts of the cell components and blood film defects from the image, we present a three-phase filtration (Bodzas, 2019, pp. 47–48).

The first phase of the three-phase filtration is focused on the removal of small objects, which is performed by the modified morphological opening operation using a disk-shaped small structuring element (Bodzas, 2019, p. 48). The modification of this operation lied in the uneven ratio between the number of iterations of the dilatation and the erosion parts of the closing operation (in particular, using the ratio 8:1). Using different iteration ratios allows the regions containing the WBCs to be preserved without a considerable reduction of the cell, and effectively removes smaller objects, such as the remaining parts of the erythrocytes and the platelets resistant to the thresholding operation. The first phase of the proposed algorithm is complemented with the second filtration step, which is based on connected component labeling followed by histogram-based filtration.

This second phase of filtration is described by the following equations, where *x* and *y* are image coordinates that belong to the set of natural numbers, *C*_*i*_ denotes the cumulative sum of the same valued pixels in the image array and *I ∈* < 0, *n* >, where n is the number of distinct gray levels in the image.

(2) $$f_{x,y}\,i=\left\{ \begin{array}{c} 1\;f_{x,y}=i\\ 0\;\mathrm{else} \end{array}\right.$$

(3) $$C_{\text{i}}=\sum_{x}\sum_{y}f_{x,y}\,\left(i\right)$$

To remove all small objects in the image, we calculate the set *S* (see Eq. 4), where each value of *i* that satisfies the condition of “being small” is included. Based on the histogram evaluation, we select a threshold value *T*_*s*_ of 4,000. Values of *i* that do not satisfy the condition are excluded.

(4) $$S_{\text{i}}=\left\{ \begin{array}{c} i\;C_{\text{i}}>T_{\text{s}}\\ 0\;e\,l\,s\,e \end{array}\right.$$

The output image *g*(*x*,*y*) is constructed from the input image *f*(*x*,*y*) in such a way that only the pixels with a nominal intensity belonging to a subpart of the set *S* are distributed to the output image, while the rest are set to 0. Thus, we ensure that the least commonly occurring intensity numbers are removed from the image.

(5) $$g_{x,y}=\left\{ \begin{array}{c} f_{x,y}\;f_{x,y}\notin S\\ 0\;e\,l\,s\,e \end{array}\right.$$

**FIGURE 2:**
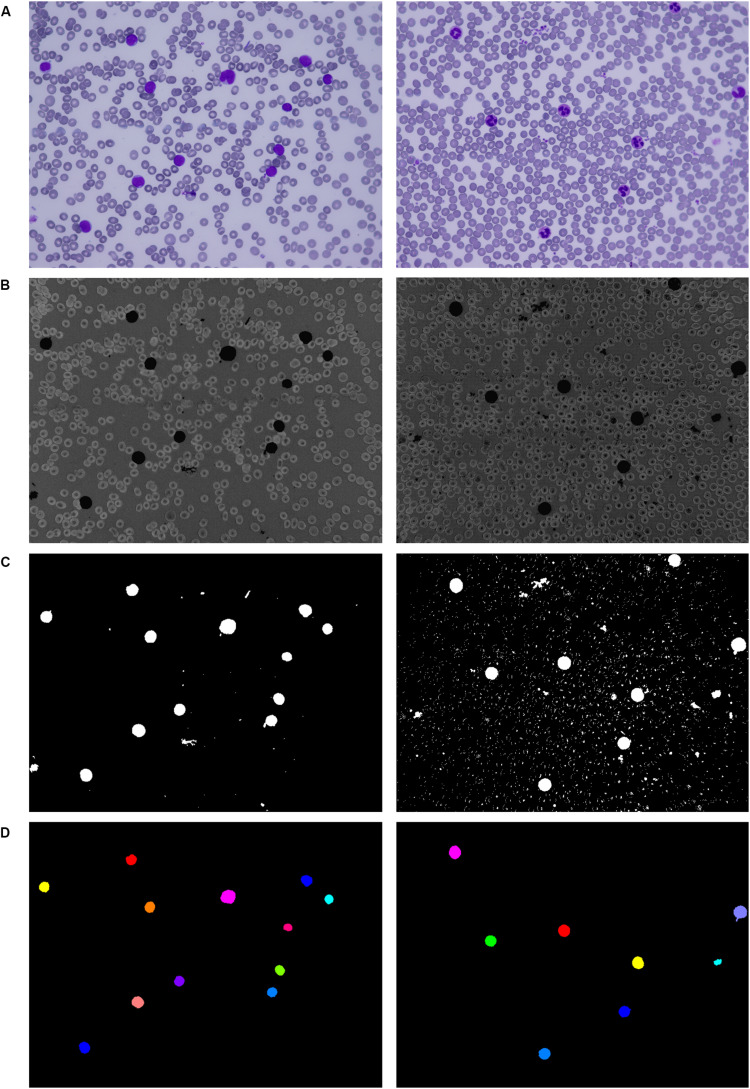
Localization of white blood cells. **(A)** Original blood smear image. **(B)** Pre-processing results. **(C)** Thresholding segmentation results. **(D)** Application of the three-phase filtration with image labeling.

Applying the second filtration step helps to smooth the image and remove all objects of small and medium size that are resistant to our opening operation. The last phase of the proposed three-phase filtration process is focused on the elimination of large blood film artifacts, which usually arise during the staining process. Since large artifacts such as precipitated stains and crushed cells have a very distinct texture and color spectrum, the mean particle color derived from the histogram is applied in combination with the particle area (Bodzas, 2019, p. 48). Using the histogram of a green color space, where the WBCs are more contrasted, prevents filtering of normal cells and cells with size abnormalities. The process of the localization of leukocytes, including the fundamental steps, is shown in Figure 2.

The blast cells tend to aggregate in clumps. The presence of such adjacent cells in an image often introduces high inaccuracy in the subsequent image processing stages. In particular, shape-based features such as the perimeter and area are highly dependent on the segmentation results. In clinical practice, to minimize the risk of miscounting, domain experts usually avoid adjacent cells or, in specific cases, solely examine clumps where the cytoplasm and nucleus are clearly identifiable. Each clearly detectable clump or adjacent cell in the image should therefore be identified and then separated into individual cells. For the identification of adjacent cells and cell clumps, the total particle area computation and morphological erosion, in combination with particle counting, are implemented. Morphological erosion is, in this case, used to separate touching objects that can be subsequently counted. Since the blast cells are nearly round and the touching edge length is smaller than the radius of either object, the touching cells can be separated well without concern that the objects will be eroded into nothing. After detecting the adjacent cells, the cells are separated by applying the Sobel edge detection technique, which specifies the approximate region of the splitting boundary (Bodzas, 2019, p. 49).

**FIGURE 3:**
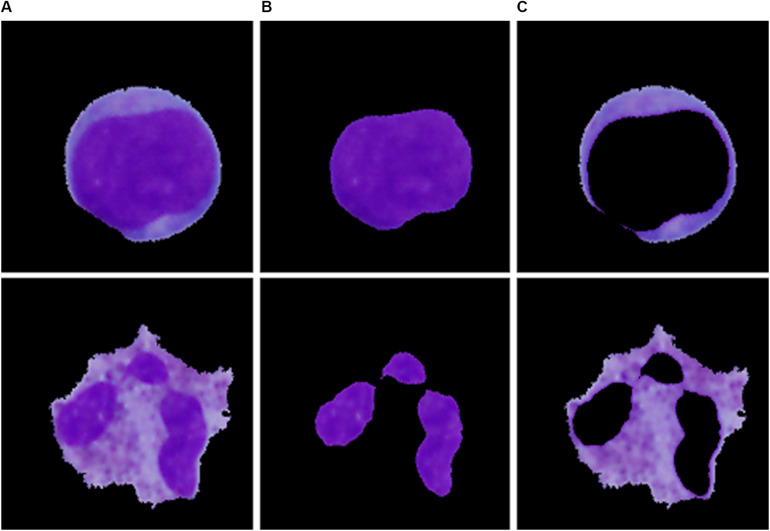
The particular segmentation results of the blast cell (top) and normal leukocyte (bottom). **(A)** Segmented cell. **(B)** Segmented nucleus. **(C)** Segmented cytoplasm.

Single-cell sub-image extraction was performed in this work by an automatic image crop using the bounding rectangle size, which is the smallest rectangle containing a particular component. Once the single leukocytes had been identified and cropped into single-cell sub-images, we finally proceeded to the second segmentation stage (region extraction), which focuses on the extraction of the nucleus and the cytoplasm into individual parts. Thus involves the following steps: nucleus localization, nucleus extraction, and extraction of the cytoplasm. To localize the nucleus, we employed equalization in the luma plane and performed color thresholding to extract the saturation channel from the HSL space, where the border of the nucleus seemed to be the most prominent. The process of nucleus extraction was accomplished by multiplying the original sub-image with the obtained binary image. Finally, the separated nucleus was used to obtain the cytoplasm by subtracting the nucleus from the original image. The results of the region extraction algorithm are shown in Figure 3.

### Features Extraction

In general, the extracted features describe the texture or shape information obtained from the segmented pattern and thereby help to reduce the dimensionality of the image to produce a result that is more informative and less redundant than the original image (Wan and Mak, 2015). In this phase, we aimed to extract the descriptive information from an image in the way that domain experts do. The proper selection of the features is considered the second most challenging step in the field of automated identification of leukemic cells. To construct an effective feature set, several published articles and their feature selection methods were studied. In this work, we implemented sixteen widely used features, of which nine had morphological characteristics and seven had statistical characteristics (Bodzas, 2019, p. 51). Another approach to extract features is the use of a convolution neural network model, which extracts a collection of feature vectors (Gao et al., 2019). In contrast to our approach, this feature space does not carry fully comprehensible information, and therefore cannot be interpreted in deep detail.

#### Morphological Features

According to hematology experts, the shape of the nucleus has proven to be a good measure for immature cell recognition. Apart from rudimentary measures such as the nucleus and cytoplasm area and nucleus perimeter, the following shape descriptors were considered.

##### Nuclear-cytoplasmic ratio

The ratio of the area of the cell nucleus to the cytoplasm area. This measure is a pivotal feature for the assessment of the maturity of the cell and, in turn, the prediction of cell malignancy. In general, the size of the nucleus decreases with increasing degree of leukocyte maturity.

##### Nucleus compactness

The extent to which the shape is compact. Depending on the maturity and the type of the WBC, the shape of the nucleus varies greatly. Mature cells usually have multi-lobed nuclei with lobes connected by thin strands or bands. Furthermore, in specific cases, the nucleus can have kidney bean or horseshoe-shaped contours. By contrast, leukemic cell nuclei are generally ovoid or round in shape and exhibit higher overall compactness than the nuclei of to mature cells. The compactness measure is given by the following formula (Chan et al., 2010).

(6) $$\mathrm{Compactness}=\frac{{\text{Perimeter}}^{2}}{\text{Area}}$$

##### Nucleus form factor

A measure of shape irregularities independent on the object’s size. In general, a circular nucleus has the greatest area to perimeter ratio, and this measure is equal to 1 for a perfect circle. Consequently, for the nuclei of leukemic cells, this ratio converges to a value of 1, while the nuclei of normal cells which depart from roundness have a lower value. The form factor is defined as

(7) $$\mathrm{Form}\,\mathrm{factor}=\frac{4^{*}\,\pi^{*}\,\mathrm{Area}}{{\text{Perimeter}}^{2}}$$

##### Nucleus eccentricity

Nucleus eccentricity indicates the deviation from a circular shape. This measure is calculated as the ratio of the length and width of the minimal bounding rectangle of the region of interest. Unlike the form factor, this measure takes into account the elliptic shapes or circular lobes of the nucleus.

##### Nucleus elongation

Nucleus elongation indicates abnormal bulging. This measure is calculated as the ratio of the maximum and minimum distance from the center of gravity to the boundary. This feature highlights WBCs with a multi-lobed elongated nucleus.

##### Nucleus solidity

Nucleus solidity defines the degree to which the shape is convex or concave and is computed as the ratio of the area and the convex hull area (Ahmed et al., 2016).

#### Statistical Features

Other indispensable descriptors used for the identification of blast cells are based on changes in the nuclear chromatin pattern reflecting DNA formation and on cytoplasmic changes. To capture the crucial information of the structural arrangement of the nucleus and the entire cell, two types of statistical measures were used. The first-order statistical measures are based on the histogram of the greyscale image, e.g., the cytoplasm and the nucleus mean color, and the second-order statistical measures are derived from the gray level co-occurrence matrix (GLCM), which carries information about the spatial relationships of the image pixels. The second-order statistical features selected in this study are defined by the equations below, where *P*(*i*, *j*) is the element of the normalized GLCM at the coordinates *i* and *j*, *N*_*g*_ denotes the number of distinct gray levels and μ_*x*_,μ_*y*_ and σ_*x*_,σ_*y*_ represent the means and standard deviations of the normalized gray level co-occurrence matrix, respectively (Bodzas, 2019, pp. 52–53).

##### Nucleus energy

A measure of the local textural uniformity of gray levels, defined as

(8) $$E\,n\,e\,r\,g\,y=\sum_{i,j=0}^{N_{\text{g}}-1}{\left(P_{i,j}\right)}^{2}$$

##### Cell contrast

Cell contrast measures the number of local variations in the GLCM. This measure is given by the relation.

(9) $$C\,o\,n\,t\,r\,a\,s\,t=\sum_{n=0}^{N_{\text{g}}-1}n^{2}\,\left\{\sum_{i=1}^{N_{\text{g}}-1}\sum_{j=1}^{N_{\text{g}}-1}P\,\left(i,j\right)\right\},\left|i-j\right|=n$$

##### Nucleus correlation

Nucleus correlation represents the linear dependency of gray tone values in the GLCM. The correlation measure is given by the following formula.

(10) $$C\,o\,r\,r\,e\,l\,a\,t\,i\,o\,n=\frac{\sum_{i}\sum_{j}\left(i,j\right)\,P\,\left(i,j\right)-\mu_{\text{x}}\,\mu_{\text{y}}}{\sigma_{\text{x}}\,\sigma_{\text{y}}}$$

##### Cell dissimilarity

Cell dissimilarity calculates the mean of the gray level difference distribution of a region and is given by the relation.

(11) $$D\,i\,s\,s\,i\,m\,i\,l\,a\,r\,i\,t\,y=\sum_{i=0}^{N_{\text{g}}-1}\sum_{j=0}^{N_{\text{g}}-1}\left|i-j\right|\,P\,\left(i,j\right)$$

##### Cell entropy

Cell entropy measures the randomness or complexity of texture. The entropy can be calculated using the following formula (Batchelor and Waltz, 2001; Nailon, 2010; Ahmed et al., 2016).

(12) $$E\,n\,t\,r\,o\,p\,y=-\sum_{i=0}\sum_{j=0}P\,\left(i,j\right)\,\log P\,\left(i,j\right)$$

All selected features were validated by using the statistical hypothesis testing method, which determined whether the samples representing the normal and blast cells came from the same population, or in other words, whether the distribution was the same for both classes. Since the analyzed data did not have a normal distribution, the median and median absolute deviation (MAD) were the proper measures to describe the observations in the dataset. In general, the analyzed features can be considered to be separable in the case of sufficiently different median values and low values of MAD that describe how spread out the data are. In this work, we used the Mann–Whitney *U* test to evaluate the statistically significant differences between the two observed groups. Table 1 shows the resulting probabilities (*p*-values) that the distributions, or in simple terms, the changes in the median values of the two classes, are not significantly different (Bodzas, 2019, p. 54).

**TABLE 1:** To show that the medians of the two datasets are different by the two-tailed Mann–Whitney hypothesis test, we employed the methodology of proof by contradiction, where the truth of a statement is determined by assuming that the null hypothesis is false.

**Features**	**Normal cell**		**Leukemic cell**		***U* test**
	**Median**	**MAD**	**Median**	**MAD**	***p*-value**
**Morphological**					
Cytoplasm area	11985.00	4894.53	4022.00	1799.24	<<0.001
Cell area	20011.00	6031.12	16255.00	2830.51	<<0.001
*N*/*C* ratio	0.75	0.21	3.15	1.15	<<0.001
Nucleus perimeter	521.00	112.64	412.00	58.51	<<0.001
Nucleus compactness	30.71	14.14	13.13	2.47	<<0.001
Nucleus form factor	0.41	0.19	0.96	0.18	<<0.001
Nucleus elongation	6.97	7.12	1.62	0.31	<<0.001
Nucleus eccentricity	0.49	0.23	0.42	0.18	0.007
Nucleus solidity	0.84	0.09	0.96	0.02	<<0.001
**Statistical**					
Nucleus energy	0.74	0.05	0.61	0.04	<<0.001
Cell contrast	1.85	0.16	1.53	0.13	<<0.001
Cell entropy	7.37	1.42	5.15	1.20	<<0.001
Nucleus correlation	0.82	0.08	0.89	0.05	<<0.001
Cell dissimilarity	0.56	0.08	0.40	0.07	<<0.001
Cytoplasm mean color	2.34	0.92	0.73	0.34	<<0.001
Nucleus mean color	0.37	0.20	0.57	0.22	<<0.001

*In our case, the defined null hypothesis states that there is no significant difference between the observed groups. The selection of a confidence level of 95% therefore signifies that the resulting p-values less than 0.05 are considered statistically significant. This indicates that there is strong evidence against the null hypothesis, as there is less than a 5% likelihood that the null hypothesis is correct.*

According to Table 1, 15 features seem to be highly unique, with great differences between the normal and leukemic cells. Even though nucleus eccentricity results with a much lower probability, this feature is statistically significant and plays an essential role in the subsequent classification phase. Owing to the high variability of the features, which encompass a wide range of cell attributes from morphological to textural, there should not be a concern of misclassification in case of blasts with variable sizes or normal cells with size-related anomalies.

### Classification

Depending on the selected classifier, the efficiency and performance of the features may vary slightly. The classification step that classifies the input data into one of the predefined classes was carried out in this work by the two most popular supervised learning algorithms, an SVM and an ANN. To achieve the best classification results, we utilized the whole range of dataset samples to determine the optimal parameters of both classifiers. The SVM as well as ANN classifiers are designed to work with the same input vector of features that we computed.

#### SVM Model Selection

SVM is a non-linear, non-parametric discriminative classifier based on the Vapnik–Chervonenkis theory. In simple terms, SVM tries to separate the data of unknown samples by finding an optimal line or hyperplane, which represents the largest margin between the classes. In the simplest two-dimensional space, this hyperplane is a line dividing a plane into two parts. Since most of the data cannot be linearly separable in a two-dimensional space, SVM projects these non-linear samples into a higher dimensional feature space by using different kernel functions (Kazemi et al., 2016). Due to this relative flexibility, SVM distinctively affords balanced predictive performance, even in studies with a limited sample size (Pisner and Schnyer, 2020).

To select an appropriate SVM classification model, we tested various kernel functions, including the most frequent linear kernel and a set of non-linear kernels, namely, Gaussian, polynomial, and radial basis function kernels. For each kernel function, we found the maximum value of the accuracy by tuning the SVM parameters using optimization techniques. To evaluate the model’s performance, we employed the 10-fold cross-validation methodology, which produced the best out-of-sample estimates with a low bias and modest variance (Bodzas, 2019, p. 59). This approach involved the random division of the dataset into 10 groups called folds of approximately equal size. During the cross-validation process, the first fold is treated as a validation set while the method is fit on the remaining ninefold. The whole cross-validation process is then repeated 10 times, and each fold is used as the validation set once (James et al., 2013). As shown by the experimental results in Table 2, the highest classification accuracy was achieved by using the polynomial kernel function.

**TABLE 2:** Cross validation accuracy of different classification models.

**Kernel function**	**Accuracy [%]**
Linear	88.38
Polynomial	98.34
Gaussian	95.02
RBS	97.51

#### Neural Network Selection

ANN is a classification technique, that uses several computing units to imitate neurons in the human brain. All units are connected with each other via a weighted link, which determines the prominence of the respective input to the output. Each neuron in a structure performs a weighted sum of all inputs and finds the output using an activation function. This activation function decides whether the information is relevant or should not pass to the subsequent unit. The whole process of learning is based on altering the values of weights and biases depending on the calculated loss function between the actual and desired output (Zayegh and Bassam, 2018).

Due to the fact that there are no specific guidelines on how to determine the optimal neural network architecture parameters, in particular the number of hidden layers and neurons, we decided to select these parameters through a trial-and-error process. During this process, several architectures with different numbers of neurons and hidden layers were tried experimentally. The number of neural units in the first and last layers depends on the number of given inputs and desired outputs. In this paper, we consider 16 input neurons, where each neuron represents one of the extracted features, and two output neurons, for the leukemic and normal classes. In this phase, we additionally split the dataset into a training and validation set in the conventional ratio of 80:20. To prevent overfitting and concentration of the neural network into one domain, we trained the neural network on randomly chosen samples. Furthermore, we used identical learning rates for each learning cycle and repeated the learning process for 50 and 500 learning iterations for each training image. The overall performance of the particular neural network models is summarized in Table 3 (Bodzas, 2019, p. 66).

**TABLE 3:** Experimental evaluation of the accuracy of different artificial neural network architectures.

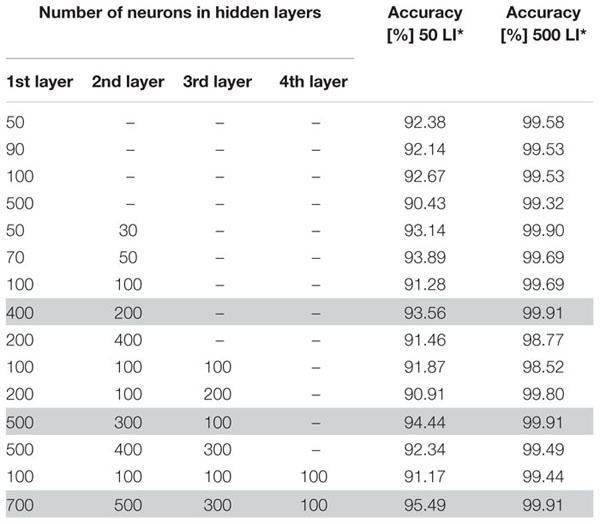			

*Learning iterations. Experimental evaluation of the accuracy of different artificial neural network architectures, with highlighted best performing setups.

The process of neural network topology verification revealed an increasing accuracy with the number of hidden layers in the case of using 50 learning iterations. We also noticed an increase of the neural network accuracy in architectures with a higher number of neurons in particular layers. On the other hand, training the neural network with a higher number of hidden layers and neurons, and 500 learning iterations, achieved greater precision and ability to classify the data correctly. In particular, the ANN models with a large difference in the number of neurons between consecutive hidden layers reached the highest accuracy, 99.91% (Bodzas, 2019, p. 68).

#### Classification Model Implementation

To perform the classification phase, we selected the best-performing models for both classifiers. Before the classification, all computed features were normalized by the min–max algorithm, which mapped the entire range of values to the range <0, 1>. For the binary SVM classification, we selected the C-SVM model, which utilizes a regularization parameter to penalize misclassifications during the separation of the classes. The best results of this classification model were achieved by applying the polynomial kernel function with a gamma value and regularization parameter of 1 and a degree parameter of 5. The tolerance of the maximum gradient of the quadratic function that was used to compute the support vectors was tuned to 0.001. In addition, to improve the functionality of this classification model, we implemented shrinking heuristics, which helped to reduce the number of variables used in the classification computation and therefore accelerated the optimization. The selected ANN model comprised two hidden layers with a descending number of neurons in particular layers (400, 200). The hidden layers of the neural network were fully connected layers without any inner modifications and utilized the sigmoid neuronal function for triggering. The initial weights for the proposed neural network were selected by the Xavier initialization process, which decreases the chance that the gradients will explode or vanish too quickly. The final process of training the architecture was performed by mean-squared error–based back-propagation and a stochastic gradient descent optimizer. Our neural network was trained with 8,333 epochs with a constant learning rate and randomly chosen samples. Moreover, during the learning process, when the measured error rate became saturated, the neural network was iteratively fine-tuned by changing the learning rate from 0.002 to 0.0001.

## Experimental Verification and Results

In the final analysis, 241 extracted sub-images of 128 normal WBCs and 113 leukemic cells were used to evaluate the proposed system. Since we have to deal with a lack of medical data, we assigned 50 percent of the dataset to the training subset, which was used to build the prediction model, and the remaining fifty percent of the data to test the proposed model. To verify the proportional distribution of specific classes between the training and testing sets, we evaluated the fundamental statistical parameters for the chosen features (see Table 4).

**TABLE 4:** The separation of the dataset into a training and testing set was performed in a way that ensured the even distribution of the whole range of blood cell types.

**Feature**	**Statistical parameter**	**Training set**	**Testing set**
Form factor	Number of samples	120	121
	Maximum value	1,21	1,21
	Minimum value	0,19	0,14
	Mean	0,70	0,73
	Standard deviation	0,30	0,32
Contrast	Number of samples	119	120
	Maximum value	3,37	3,35
	Minimum value	2,17	2,13
	Mean	2,67	2,64
	Standard deviation	0,24	0,25

*To verify that the split did not affect the statistical distribution, the maximum, minimum, mean, and standard deviation were compared between the two sets. Since all the statistical parameters of the two selected features seem to be well balanced, the final classification should not be burdened with significant errors.*

Each output of the selected classifier in this work, presents a particular probability, with which the cell belongs to the leukemic and normal class. Since the output probabilities given by the SVM model take into account only the probability of the corresponding class, we computed the absolute complement of the outputs to obtain an inversely proportional set. To assess the outputs of both classifiers, the winner-take-all principle was implemented in the last phase. This means that only the classification outputs with the highest score were considered to be the final results. The performance of both algorithms was subsequently estimated by constructing the confusion matrices for both implemented classifiers (see Table 5).

**TABLE 5:** Summarization of all correct and incorrect classifications.

	**SVM**		**ANN**	
	**Disease positive**	**Disease negative**	**Disease positive**	**Disease negative**
**Test positive**	56	3	57	3
**Test negative**	1	61	0	61
	Overall accuracy: 96.72%		Overall accuracy: 97.52%	

Namely, the specificity, sensitivity, accuracy, F1 score and error rate metrics of the proposed strategy were assessed using the following formulas, where TP stands for the number of true positives, TN stands for the number of true negatives and FP and FN denote the numbers of first and second error types (false positives and false negatives, respectively) (Chen et al., 2019).

(13) $$A\,c\,c\,u\,r\,a\,c\,y=\frac{\mathrm{TN}+\mathrm{TP}}{\mathrm{TP}+\mathrm{FN}+\mathrm{FN}+\mathrm{FP}}\,$$

(14) $$S\,e\,n\,s\,i\,t\,i\,v\,i\,t\,y=\,\frac{\text{TP}}{\mathrm{TP}+\mathrm{FN}}\,$$

(15) $$S\,p\,e\,c\,i\,f\,i\,c\,i\,t\,y=\,\frac{\text{TN}}{\mathrm{TN}+\mathrm{FP}}\,$$

(16) $$F_{1}=\,\frac{{\text{Sensitivity}}^{*}\,\,\text{Specificity}}{\text{Sensitivity}+\text{Specificity}}=\frac{2\,T\,P}{2\,T\,P+\text{FP}+\text{FN}}$$

(17) $$E\,R\,R=\frac{\text{FP}+\text{FN}}{\text{TP}+\text{FP}+\text{TN}+\text{FN}}\,$$

The sensitivity and specificity represent warnings from two different standpoints. Whereas sensitivity indicates how often positive predictions are correct, specificity denotes the percentage of successful negative predictions. In the medical field, reaching 100% specificity is not reasonable. This value of this type of measure is reached in medical practice by the assumption that no patients have a positive diagnosis and that therefore, the test will never make an FN error. However, high values of specificity are required in cases where the main goal is to limit the number of false negatives. To achieve a better overview of diagnostic efficiency, we took into account the F1 score metric, which combines both sensitivity and specificity (Tharwat, 2018). Table 6 shows the comparison of the implemented classifiers in terms of their prediction performance (Bodzas, 2019, p. 70).

**TABLE 6:** Performance measures for selected supervised classifiers.

	**Accuracy**	**Sensitivity**	**Specificity**	***F*_1_**	**Error rate**
SVM	96.72	98.25	95.31	96.55	3.28
ANN	97.52	100.00	95.31	97.44	2.48

Examples of specific classification results highlighting all incorrectly classified cells are presented in Table 7. Two cases of incorrect classifications were caused by a flawed segmentation phase (incorrectly classified cells D and E). Nevertheless, the ANN, due to its ability to accept relatively small errors, identified one of those cells correctly with an accuracy of 98.19%. Even though the ANN proved to have a better performance, in the case of the incorrectly classified cell C, we notice overfitting, which is the major drawback of this methodology. On the contrary, overfitting is not seen in the results obtained by the SVM algorithm, which achieved better identification results in this sample. The main reason lies in the evenly distributed portions of similar cells among the learning and training sets and the small degree parameter, which decreased the flexibility of the decision boundary and therefore prevented overfitting. Other practical problems are often caused by missing image samples in the datasets. Such missing samples in the training set are sometimes indispensable for making correct predictions. This can be seen in case B among the incorrect classifications, where the lack of banded neutrophils resulted in an accuracy of 0% for both classifiers. Whereas all incorrect ANN classifications were related to the first kind of error, of predicting a positive diagnosis when the actual condition was negative, the SVM in one sample (A) resulted in the worst-case scenario (a type II error) by predicting disease absence.

**TABLE 7:** The classification probabilities of selected samples.

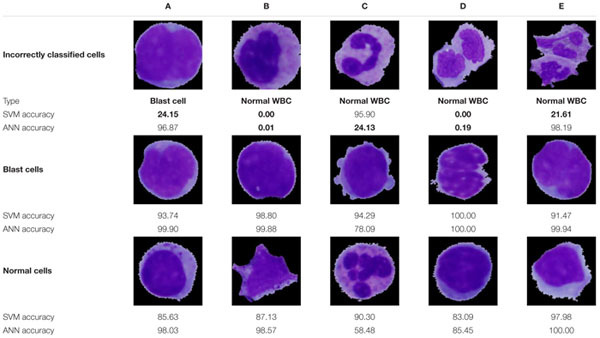

*The first four rows (A–E) show examples of all incorrectly classified samples with the false positive and false negative classifications highlighted, and the rest of the rows (A–E) show the probability results of the selected examples of correct classifications.*

It should also be noted that even though the remaining cells were classified correctly, some results do not achieve a classification probability higher than 95%, and therefore, there is a high probability of the presence of overfitted areas in the vicinity of these cells.

## Conclusion and Future Prospects

In this work, we propose a method for the automated identification and classification of blast cells from microscopic peripheral blood smear images. This study introduces a novel combination of image processing methodologies and proposes extensive pre-processing to achieve high classification accuracy. In particular, the selected combination of 16 features carrying morphological and statistical information demonstrated an excellent ability to distinguish between cancerous and non-cancerous blood cells. We selected most of the features on the basis of their similarity with the visual information, on which the domain experts focus during manual examination. These features were extracted from 241 WBCs segmented from 31 peripheral blood smear images from a local dataset. To perform the classification, we selected the two most popular classifiers in the literature, the ANN and the SVM algorithm. The neural network model yielded better results, reaching a sensitivity of 100% and an overall accuracy of 97.52%. Unlike previous studies, we also presented some of the specific classification probabilities of the correctly identified cells and conducted a reverse analysis to identify the pivotal classification failures. These observations indicated that even when the published accuracies reach the highest values, a classification method may not provide clarity or sufficiently high reliability, and therefore, further examination is required.

One of the greatest problems we encountered was a lack of medical data and extensive datasets. In particular, expanding the learning set of the data would reduce overfitting and increase the probability of particular classifications. Moreover, the classification errors caused by incomplete datasets with missing cell samples would be suppressed. It should be noted that many authors have verified their proposed systems by employing small local and publicly unavailable datasets. Due to this fact, it was impossible to compare our findings with the results obtained by the previously proposed algorithms. Furthermore, this has a negative impact on the possibility of reproducing recent trends and converging toward better technical solutions. The results obtained in this work indicate that future research should be mainly devoted to the development of a more robust segmentation algorithm with the possibility of adaptive parameter adjustment, which would unify the functionality of the system under diverse conditions. Moreover, researchers should focus on improving particular classification probabilities and minimizing false negative classifications. Such a system could be then used as a medical support tool that would facilitate manual examination and save tremendous time. Using the results of particular classifications with a defined high decision limit will allow us to achieve higher identification reliability. Nevertheless, cells with lower probability should be still verified by hematological specialists.

## Data Availability Statement

The datasets generated for this study are available on request to the corresponding author.

## Ethics Statement

The studies involving human participants were reviewed and approved by the Ethics Committee of FN Ostrava, University Hospital Ostrava. The participants provided written informed consent to participate in this study.

## Author Contributions

AB conceived and designed the study and drafted the manuscript. JZ coordinated the study and provided useful suggestions. PK performed searches, analyses, interpretations, and edited the manuscript. AB and PK developed machine learning algorithms. All authors contributed to the article and approved the submitted version.

## Conflict of Interest

The authors declare that the research was conducted in the absence of any commercial or financial relationships that could be construed as a potential conflict of interest.
